# Immunotherapy and Radiation for Clinical Perineural Invasion in Cutaneous Squamous Cell Carcinoma

**DOI:** 10.3390/cancers17243921

**Published:** 2025-12-08

**Authors:** Renee A. Morecroft, Jordan S. Phillipps, Lang Gou, Alok A. Bhatt, Sungjune Kim, Homan Mohammadi, Roxana S. Dronca, Bently Doonan, Ruqin Chen, Yujie Zhao, Hye Seon Kang, Shenduo Li, Jeffrey R. Janus, Phillip Pirgousis, Samip Patel, Oluwafunmilola T. Okuyemi, Elisha M. Singer, Leila M. Tolaymat, Ashley Wysong, Catherine A. Degesys, Naiara Barbosa, Adam L. Holtzman

**Affiliations:** 1 Department of Internal Medicine, HCA Florida Orange Park Hospital, Orange Park, FL 32073, USA; 2 Department of Dermatology, Mayo Clinic, Jacksonville, FL 32224, USA; 3 College of Chemistry, UC Berkeley, Berkeley, CA 94720, USA; 4 Department of Radiology, Mayo Clinic, Jacksonville, FL 32224, USA; 5 Department of Radiation Oncology, Mayo Clinic, Jacksonville, FL 32224, USA; 6 Division of Hematology and Medical Oncology, Mayo Clinic, Jacksonville, FL 32224, USA; 7 Division of Pulmonology, Department of Internal Medicine, Bucheon St. Mary’s Hospital College of Medicine, Seoul 14647, Republic of Korea; 8 Department of Otorhinolaryngology/Audiology, Mayo Clinic, Jacksonville, FL 32224, USA

**Keywords:** cutaneous oncology, head and neck cancer, immunotherapy, perineural invasion, radiotherapy, skin cancer, squamous cell carcinoma

## Abstract

Localized cutaneous squamous cell carcinoma (cSCC) has a favorable prognosis, unlike advanced disease, especially with clinical perineural invasion (PNI), which poses substantial management challenges due to aggressivity and higher recurrence, metastasis, and mortality risks. PNI, a high-risk staging feature, has worse outcomes, particularly when clinically evident rather than incidental. Clinical PNI (cPNI) is evident by clinical symptoms (such as pain, paresthesia, or motor deficits) or radiologic findings, whereas incidental PNI (iPNI) is identified only histologically without associated symptoms or radiologic evidence. PNI remains a novel area with varying practice patterns across institutions. Improving risk stratification and tailoring multidisciplinary approaches are critical for optimizing outcomes. Our review outlines clinical practice patterns at our institution, providing insights into managing cSCC with PNI, focusing on diagnosis, imaging, staging, and emerging immunotherapies. A structured search was conducted using the terms “perineural invasion,” “cutaneous squamous cell carcinoma,” and “immunotherapy.” cPNI has a poor prognosis and requires nuanced clinical decision-making. Surgery and radiation remain central to management. Adjuvant therapy offers substantial survival benefit in cSCC with PNI, with improved disease-free and overall survival compared with surgery alone, supporting its use in appropriately selected high-risk patients. Traditional systemic therapies, including cisplatin and cetuximab, remain foundational but have shown only moderate response rates and limited durability in advanced or neurotropic cSCC. In contrast, immunotherapy—now preferred for advanced or unresectable cases—has transformed management, with programmed cell death protein-1 (PD-1) inhibitors showing promising results (up to 69% response rate) and disease stabilization. Neoadjuvant immunotherapy may enable tumor downstaging, improve radiation planning, and reduce surgical morbidity. Imaging for squamous cell carcinoma (SCC) with PNI aids staging and surveillance, but symptoms remain key for detecting recurrence. Our multidisciplinary approach emphasizes personalized care. Larger trials are needed to define the optimal role and sequencing of immunotherapy in this high-risk patient population.

## Simple Summary

We highlight the growing role of immunotherapy in treating high-risk cutaneous squamous cell carcinoma with clinical or radiographic nerve involvement, which is often difficult to manage with surgery or radiation alone. This work explores how using immunotherapy before or in place of traditional treatments may shrink tumors, improve symptoms, and reduce the need for extensive procedures. We also examine how combining immunotherapy with advanced radiation techniques may enhance tumor control while limiting side effects. By emphasizing the importance of coordinated care among dermatologists, surgeons, oncologists, and radiologists, we aim to support more personalized and effective multidisciplinary treatment strategies based on institutional experience. These insights may encourage a broader shift toward immunotherapy-based approaches and help the research community refine care for patients with these complex cases.

## 1. Background

### 1.1. Epidemiology

SCC is the second most common skin cancer, accounting for 20% of keratinocyte carcinomas [1]. As our population ages, the incidence of SCC is anticipated to increase and already represents more than 1 million new cases annually. Therefore, increasing associated treatment costs and patient morbidity highlight the need for early, accurate diagnosis and efficient risk stratification to optimize management. The prognosis for localized SCC is generally favorable, with high cure rates following surgical excision [1]. Advanced SCC, which may not be amenable to surgery or radiation therapy (RT), often exhibits aggressive features such as deep invasion, poor differentiation, and PNI [2]. Notably, PNI in SCC is associated with increased rates of recurrence, metastasis, and disease-specific mortality [2].

### 1.2. Risk Factors, Staging, and Evaluation

Key risk factors for the development of SCC include advanced age, immunosuppressive states (chronic lymphocytic leukemia, solid organ/stem cell transplant, immunosuppressive medications), and chronic inflammatory states (burn scars and inflammatory dermatoses) [1]. Tumor characteristics associated with poor outcomes include poor differentiation, large diameter (>2 cm), increased depth of invasion (>6 mm), lymphovascular invasion, and PNI [3,4]. SCC staging systems, such as those from the American Joint Committee on Cancer (AJCC) and Brigham and Women’s Hospital (BWH), highlight the prognostic significance of PNI [1]. The AJCC defines PNI as tumor invasion of nerves measuring 0.1 mm or greater in diameter or depth below the dermis [1]. Histologic evidence for PNI requires demonstration of tumor cells within any of the three layers of the nerve sheath (epineurium, perineurium, or endoneurium), or in close association with at least one-third of the nerve circumference [5]. Additional high-risk features of SCC include tumor thickness greater than 2 mm, Clark level IV/V, poor histologic differentiation, and anatomic location on high-risk sites (e.g., lips and ears) [6]. Notably, the AJCC and BWH staging systems upstage tumors with PNI based on the presence of other high-risk features. Consequently, evaluation requires a thorough clinical history, detailed histologic assessment, physical examination, and appropriate imaging studies. Patient interview should focus on identifying risk factors for PNI, such as neuropathic pain or paresthesia [6]. Histologic review should assess for PNI and other high-risk features, which are essential for accurate staging. Physical examination should document lesion size, location, and clinical features. Imaging modalities such as magnetic resonance imaging (MRI) and computed tomography (CT) are valuable for assessing local invasion and detecting PNI [6].

### 1.3. Management

Management of SCC typically involves surgical resection with larger surgical margins or Mohs Micrographic Surgery due to its precision in margin control and tissue conservation (especially for head/neck tumors) [6]. RT is an important option for patients who are poor surgical candidates—particularly for tumors in the head and neck, where surgery often requires extensive tumor resection and neck dissection—or as adjuvant treatment for high-risk SCC, including lesions with clinical, extensive small-caliber, or named-nerve PNI [6].

Advanced SCC can metastasize to regional lymph nodes. Radical lymph node dissection is the standard surgical approach for managing regional nodal involvement. Adjuvant RT is recommended postoperatively for high-risk features, such as extracapsular nodal extension or positive surgical margins, improving locoregional control and overall survival [6].

The role of traditional systemic chemotherapy in advanced SCC is limited, and it could be argued that it does not have a significant role in the era of immunotherapy (though management paradigms should be appropriately individualized) [1]. Prior to immunotherapy, cisplatin was commonly used, either as monotherapy or in combination with other chemotherapeutic agents [1]. High-dose cisplatin (100 mg/m^2^ every 21 days) was typically administered concurrently with RT for its synergistic effects, although lower weekly doses (40 mg/m^2^) may be used to minimize toxicity [7]. Cetuximab, an epidermal growth factor receptor inhibitor, has demonstrated improved locoregional control and survival when combined with RT, particularly in patients ineligible for cisplatin-based therapy. However, emerging evidence at the time suggested that cetuximab may be less effective than cisplatin in certain subgroups, such as those with human papillomavirus–positive oropharyngeal cancer, highlighting the need for individualized risk stratification and management [7]. Of note, studies often included both cSCC and head and neck (HNSCC), despite their differing biology and treatment responses, which limits the applicability of chemotherapy data from HNSCC to cSCC [7]. Chemotherapy and epidermal growth factor receptor inhibitors remain options for persons who are ineligible for immunotherapy, but responses are generally less effective. More recently, immunotherapy has been approved for use in locally advanced, recurrent, or metastatic cSCC (particularly those ineligible for curative surgery/RT); however, little is known about the utility of immunotherapy in SCC patients with clinical PNI. PD-1 inhibitors (e.g., Cemiplimab, pembrolizumab, Cosibelimab, etc.) are specifically noted as recommended systemic monotherapy in such instances, with higher response rates and less toxicity over conventional chemotherapies/targeted therapies [1].

Overall, effective management of advanced SCC requires a multidisciplinary approach, involving dermatologists, oncologists, surgeons, and radiologists, to develop personalized treatment plans that optimize patient outcomes. Use of immunotherapy in treating PNI-associated cSCC remains a novel area with varying practice patterns across institutions, highlighting a clinically relevant knowledge gap. The aim of our review was to summarize the evolving literature and our institution’s current multidisciplinary clinical approach to managing this complex disease.

### 1.4. Perineural Invasion

PNI occurs in approximately 2% to 6% of keratinocyte carcinomas, more frequently in SCC than in basal cell carcinoma [8,9]. Risk factors for PNI include male sex, tumor recurrence, midface location, poor differentiation, and deep subclinical extension [10]. PNI increases the risk of tumor recurrence, metastasis, and poor prognosis, especially when involving large-caliber (≥0.1 mm) or named nerves and extending beyond the dermis. Conversely, small-caliber (<0.1 mm) nerve involvement and superficial invasion confer more favorable outcomes [5,10]. Notably, skip lesions along nerves may result in recurrence and false-negative histopathologic margins [8].

PNI is classified as iPNI or cPNI. iPNI is detected histologically in the absence of neurologic symptoms or imaging findings and is generally asymptomatic [8]. cPNI, seen in about one-third of cases, presents with neurologic symptoms (e.g., pain, numbness, facial weakness) or radiologic evidence of nerve involvement (often affecting the trigeminal/facial nerves) and indicates more extensive disease [8,9]. cPNI has a worse prognosis than iPNI, with lower 5-year control rates (55% vs. 80%) and higher disease-specific mortality (75% vs. 65%) [10]. Extensive PNI (involvement of ≥5 nerves) has been reported, though its prognostic impact remains unclear [5,11]. Given its prognostic significance, the presence of PNI upstages tumors in staging systems such as those from the AJCC and BWH [5].

The location of SCC significantly affects the risk of developing PNI, with tumors in the head and neck region—especially the temple, ear, lip, periorbital area, tongue, and floor of mouth—demonstrating a higher incidence [12]. The underlying reason is multifactorial. Anatomic proximity to larger, named nerves and higher nerve density in these regions facilitate tumor access to neural structures [13]. Molecular crosstalk between tumor cells and nerves—including neurotrophin signaling—may further promote PNI in head and neck SCCs [14]. Histopathologic features such as poor differentiation, desmoplasia, and increased tumor thickness, which are more common in high-risk locations, also contribute to PNI risk [15].

Management strategies vary based on PNI type. While PNI is a high-risk feature for SCC, iPNI can be classified as lower risk (if small caliber or in the dermis and not multifocal), where cases may be managed by surgery alone, with adjuvant RT reserved for additional high-risk features such as immunosuppression, tumor size of 2 cm or larger, or poor differentiation [16]. cPNI is always considered a higher risk and is typically treated with surgery followed by postoperative RT or definitive RT if inoperable [9,16]. While chemotherapy may improve local-regional disease control in high-risk SCC with PNI, its routine role remains unestablished [8]. Notably, current treatment guidelines do not specifically recommend immunotherapy for PNI; therefore, our practice patterns may influence future management, particularly in patients with cPNI at increased risk of recurrence, complications, and mortality.

## 2. Immunotherapy in SCC

### 2.1. Evidence for Immunotherapy

Immunotherapy with immune checkpoint inhibitors (ICIs) has been approved for the treatment of unresectable, locally advanced, or metastatic SCC [17]. We have summarized our literature review in Table 1, and a diagram outlining our search methodology has been included as Figure S1 [18,19,20,21,22]. Several databases were searched using the keywords “perineural invasion,” “immunotherapy,” and “cutaneous squamous cell carcinoma.” Cemiplimab, an anti–PD-1 agent, received US Food and Drug Administration approval based on the phase 2 EMPOWER cutaneous SCC (cSCC)-1 trial and a phase 1 trial (NCT02383212) for patients with locally advanced or metastatic cSCC ineligible for curative surgery or RT [17]. In the phase-3 C-POST trial, adjuvant cemiplimab has also demonstrated significantly prolonged disease-free survival with marked reductions in both locoregional and distant recurrence in resected cSCC post-surgery and radiotherapy. Though histologic PNI was an explicit eligibility criterion, outcomes have not yet been reported in a PNI-stratified subgroup analysis [23]. A retrospective analysis of 13 patients with head and neck cSCC and cPNI treated with anti–PD-1 therapy reported an objective response rate of 69.2% (46.2% complete response and 23.1% partial response) (Table 1) [21]. Of this cohort, 3 (23%) experienced progressive disease, with a median time to progression of 3.5 months (Table 1) [21]. Meanwhile, 9 patients (69.2%) achieved an objective response (complete or partial), with a median time to response of 2.1 months (Table 1) [21]. No grade 3–4 treatment-related adverse events were observed (Table 1) [21]. These findings suggest that while a majority of patients responded to therapy, a subset progressed relatively early during treatment. Additionally, a systematic review highlighted the efficacy of anti–PD-1 therapy in cutaneous malignancies with cPNI, with 61.2% of patients achieving either complete local response or stable disease (Table 1) [19]. These findings reinforce the role of ICIs as a viable treatment option for patients with cSCC and cPNI, especially when surgery and RT are not feasible.

Neoadjuvant immunotherapy with PD-1 inhibitors, such as cemiplimab, has also shown promise in managing keratinocyte carcinoma. In a retrospective review by Wu et al., 11 patients with cSCC and cPNI involving large-caliber or named nerves were treated with ICIs [22]. Radiographic PNI control was achieved in 9 patients (81.8%), with 8 showing improvement and 1 demonstrating stable disease. However, complete resolution of radiographic PNI was rare, occurring in only 1 patient. Clinically, 7 patients (63.6%) showed a response, with radiographic improvement correlating well with symptomatic relief. Improvement in neuropathic pain was the most sensitive clinical marker of response [22]. These results compare favorably with earlier data on ICIs for locally advanced and metastatic cSCC, which reported objective response rates of 44% for cemiplimab, 34.3% for pembrolizumab, and up to 47% for cosibelimab [24]. Overall, neoadjuvant immunotherapy may reduce the need for extensive surgeries or adjuvant therapy, thereby minimizing patient morbidity. Other targeted therapies have been explored but have shown modest efficacy compared to ICIs. For example, cetuximab has been used in advanced cases but with limited success [25].

The most relevant predictive biomarkers for cSCC, particularly in cases with PNI, include tumor programmed death-ligand 1 (PD-L1) expression, the presence and phenotype of tumor-infiltrating lymphocytes (especially CD8^+^ T cells expressing immune checkpoints), and gene expression signatures associated with immune activation [26,27,28]. However, PD-L1 status alone does not reliably predict treatment response in cSCC [26,27,28]. Higher expression of immune activation signatures—such as major histocompatibility complex I, T-cell, natural killer-cell, interferon-gamma, and antigen-presentation pathways—is associated with better responses to PD-1 inhibitors, whereas stromal, angiogenic, and hypoxic signatures correlate with treatment resistance [28]. These findings suggest that gene expression profiling may help stratify patients, although it is not yet part of standard clinical practice. The ENLIGHT-DP biomarker, derived from transcriptomic analysis of Hematoxylin and Eosin slides, has shown promise in predicting response to PD-1 inhibition in advanced cSCC, including in patients previously treated with surgery or radiotherapy, but it has not been validated for routine use [29]. Overall, no single biomarker has been confirmed for standard use in selecting immunotherapy candidates with cSCC and PNI.

### 2.2. Role of RT

**Figure 1:**
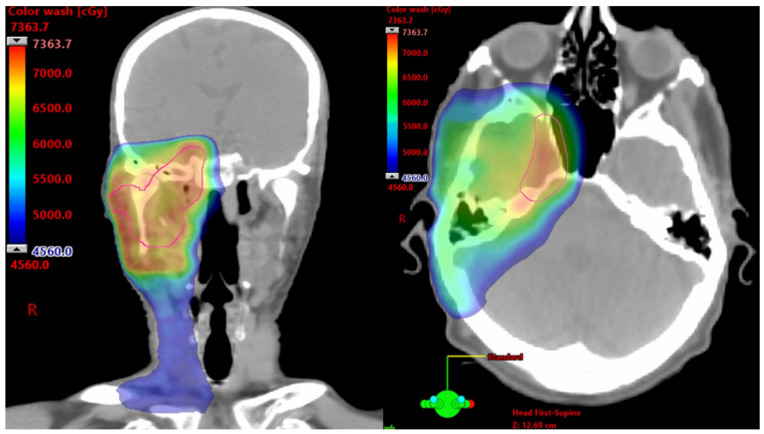
Axial and Coronal Images From a Computed Tomography Planning Scan. Color wash dose distribution of a patient receiving 7320 cGy using a 120 cGy twice/day hyperfractionated approach to the right skull base and elective right neck.

In patients with unresectable SCC and cPNI, RT has demonstrated durable local control rates exceeding 50%, even in advanced T4 disease [8,9]. However, much of this data predates immunotherapy. At some academic tertiary centers, neoadjuvant immunotherapy is now the preferred initial approach for patients with inoperable SCC and those who are poor surgical candidates due to comorbid conditions, functional limitations, or cosmetic concerns. This strategy promotes tumor shrinkage, organ preservation, and reduced toxicity from high-dose RT, particularly when tumors are near critical neurovascular structures, such as the brainstem or optic pathways. Figure 1 shows color wash dose distribution for a patient receiving definitive RT with volumetric modulated arc therapy after neoadjuvant cemiplimab. The patient showed a notable therapeutic response, with a reduction in gross disease along the third division of the trigeminal nerve to its root at the brainstem. This response enabled delivery of high-dose conformal RT while adhering to organ-at-risk constraints. Altered fractionation with volumetric modulated arc therapy and highly conformal RT techniques are typically used to mitigate acute and late toxicities. Nonetheless, given the proximity to critical structures and the extent of disease, complete elimination of risk remains challenging [30].

### 2.3. Synergistic Immunomodulatory Effects of RT

As noted above, RT can achieve strong locoregional control in unresectable and locally advanced disease; however, its benefits may extend beyond local effects [10,31]. RT complements immunotherapy by priming the tumor microenvironment [10,31]. It induces immunogenic cell death, promotes tumor antigen presentation, activates innate immunity, and recruits effector T cells. Mechanistically, RT triggers the release of damage-associated molecular patterns, which activate antigen-presenting cells to promote T-cell priming. It also upregulates chemokines, facilitating immune cell infiltration—a key consideration as immunotherapy becomes standard in cSCC [32]. Preclinical work by Dovedi et al. demonstrated that combining RT with PD-1/PD-L1 blockade synergistically improves tumor control and survival, with long-term complete responses in up to 80% of treated mice (*P* < 0.001) compared to RT alone [33]. Survivors developed antigen-specific memory T-cell responses capable of rejecting tumor rechallenge [33]. Clinically, Ferini et al. noted that concurrent RT (particularly hypofractionated) and immunotherapy may produce durable responses in locally advanced cSCC, with noted potential for metastatic cSCC [31].

Retrospective studies suggest improved outcomes in advanced cSCC when RT is added to systemic therapy, with one showing median overall survival of 13 months vs. 7 months for systemic therapy alone. Similar benefits have been observed in head and neck oropharyngeal SCC [31]. For example, the Quad Shot regimen with ICIs yielded 85% 12-month local control versus 63% with RT alone [34]. However, clinical data are still evolving. Studies have indicated that high PD-L1 expression and abundant CD8^+^ tumor-infiltrating lymphocytes are associated with improved survival and response to chemoradiotherapy. Conversely, a phase II trial found that adding stereotactic body RT to nivolumab in metastatic HNSCC did not improve response rates compared to nivolumab alone [35].

Particle therapy, including proton and carbon ion radiation, may further enhance immune stimulation more effectively than conventional photon therapy. The distinct Bragg peak of particle therapy allows for greater precision and better sparing of normal tissue [36,37]. By minimizing exposure of healthy tissues and immune cells, particle therapy reduces the risk of treatment-induced lymphopenia [38]. Additionally, carbon ion therapy has a higher linear energy transfer, causing greater DNA damage and immunomodulatory effects, which contribute to a more proinflammatory tumor microenvironment [38,39]. Furthermore, carbon ion therapy decreases immunosuppressive cells (e.g., regulatory T cells and myeloid-derived suppressor cells), enhancing the infiltration of cytotoxic T cells, natural killer cells, and macrophages, while improving the systemic tumor immune environment by preserving lymphocyte function and reducing immunosuppressive cytokines [40]. A recent preclinical study demonstrated that combining carbon ion therapy with ICIs yielded positive abscopal responses and survival [40]. Ongoing research is exploring how particle therapy can further strengthen the immune response and synergize with immunotherapy.

Although preclinical and early clinical data are promising, prospective trials are needed to define optimal RT dosing, sequencing, and patient selection in combined RT-immunotherapy strategies.

## 3. Imaging

### 3.1. Role of Imaging

Baseline imaging is essential for staging SCC with PNI, as it defines the extent of the primary tumor, evaluates nodal involvement, and may aid in detecting distant metastases. MRI and CT provide complementary diagnostic information; MRI is superior for detecting PNI (94.4% sensitivity) and intracranial extension, while CT is preferred for assessing bone involvement (sensitivity 57.5%, specificity 98.6%) and nodal disease (sensitivity 96.4%, specificity 100%) [1,41]. These imaging modalities are vital for identifying high-risk features, such as larger tumor size, increased depth of invasion, and PNI, which directly influence prognosis and treatment planning [1,42]. Clinical examination for motor or sensory deficits can help guide imaging protocols and the radiologist’s search patterns [43].

**Figure 2:**
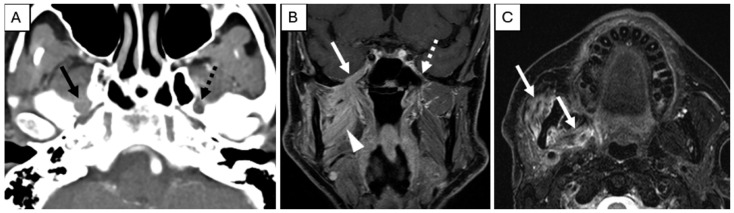
Pre-treatment Examination: Perineural Tumor Spread. (**A**), Axial contrast-enhanced computed tomography of the skull base demonstrates loss of perineural fat and thickening of the mandibular division of the right trigeminal nerve (solid arrow). Note the preserved fat plane on the left (dashed arrow). (**B**), Coronal T1-weighted, fat-saturated postcontrast image demonstrates abnormal thickening and enhancement of the right mandibular division of the trigeminal nerve extending through the foramen ovale into the cavernous sinus (solid arrow). Note the normal left side (dashed arrow). Abnormal enhancement of the right masticator space muscles (arrowhead) is compatible with subacute denervation changes. (**C**), Axial T2-weighted image demonstrates hyperintense signal within the right muscles of mastication (arrows) compatible with subacute denervation changes, directing the search pattern to the mandibular division of the trigeminal nerve.

On imaging, PNI appears as abnormal thickening or enhancement of cranial nerves and their branches (which may be discontinuous), loss of normal perineural fat planes (most often seen at the foramina), and foraminal widening at key skull base locations, including foramen rotundum, foramen ovale, cavernous sinus, and Meckel’s cave (Figure 2) [43,44,45]. In advanced disease with cPNI or nodal involvement, fludeoxyglucose F18 positron emission tomography (FDG-PET) is valuable for assessing regional and distant metastases and may show linear or focal uptake along the involved cranial nerves or foramina [43]. Imaging is particularly recommended for high-risk cSCC, including BWH stage T2b/T3 tumors, PNI, and tumors located in anatomically complex regions (e.g., head and neck) where preoperative imaging can impact therapeutic planning. Retrospective studies suggest that baseline imaging of draining nodal basins in BWH stage T2b or higher tumors reveals abnormal findings in 59% to 65%, resulting in changes to clinical management in up to 33% of patients [1,46].

### 3.2. Recommendations for Surveillance Imaging

**Figure 3:**
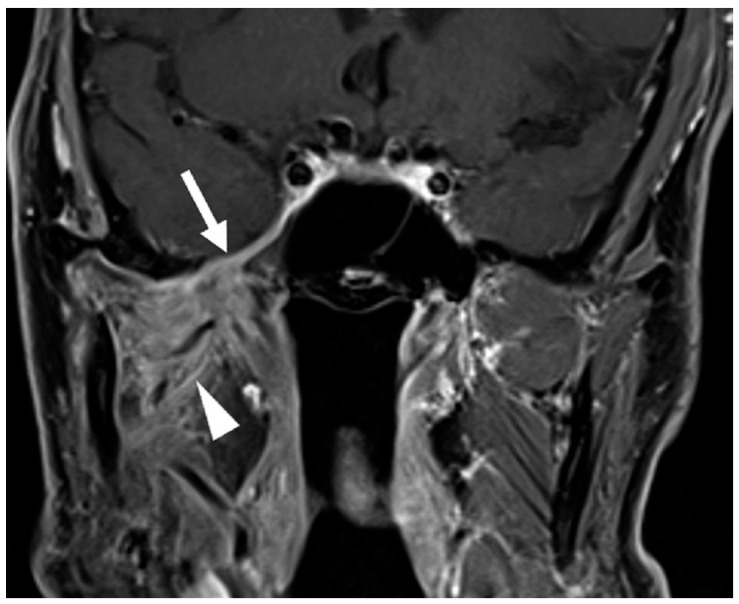
Post-treatment Examination Following Neoadjuvant Immunotherapy and Definitive Radiotherapy. Coronal T1-weighted, fat-saturated postcontrast image demonstrates decreased thickening and enhancement of the mandibular division of the right trigeminal nerve through the foramen ovale and right cavernous sinus (arrow, compared to Figure 2B). Note persistent abnormal enhancement of the right muscles of mastication (arrowhead) consistent with denervation changes; atrophy of the muscles did not resolve post-treatment.

Post-treatment imaging evaluation of perineural tumor spread presents unique challenges. Changes such as denervation and foraminal widening may persist despite an effective treatment response. Indicators of response include decreased nerve thickening or enhancement on MRI and CT (Figure 3) and reduced metabolic uptake on FDG-PET [18,45]. However, radiographic findings may not fully correlate with disease activity; thus, clinical symptoms such as worsening pain and numbness remain the most reliable indicators of recurrence or progression [47].

Imaging is recommended 8 to 12 weeks after definitive therapy to evaluate treatment response and establish a baseline for surveillance. PET-CT is preferred for its ability to provide both metabolic and anatomic detail, particularly in patients with T3/T4 or N2/N3 disease [48,49]. MRI is reserved for cases involving the skull base or PNI, where soft tissue resolution is crucial [48,49,50].

Surveillance protocols should be individualized based on recurrence risk, which is influenced by initial disease staging, human papillomavirus status (particularly for oropharyngeal SCC), and tobacco use. Surveillance imaging is crucial in high-risk patients, as 95% of asymptomatic recurrences occur within the first year after treatment [48] Standard practice includes imaging every 3 to 6 months for the first 2 to 3 years, followed by annual imaging thereafter. Adjustments may be needed for patients with high-risk features or tumors in areas not easily accessible to clinical examination [1,50,51]. Routine surveillance of PNI beyond 1 year of annual follow-up is generally of limited value unless new clinical concerns arise; however, often these tumors will need 2 to 3 years of lymph node surveillance as they are also at high risk for regional metastasis [48,50].

## 4. Multidisciplinary cPNI Management

**Figure 4:**
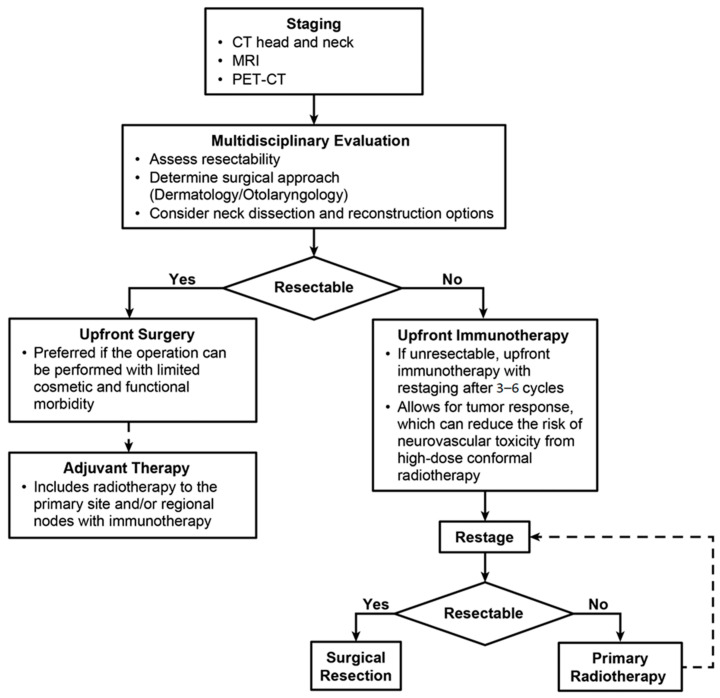
Practice Patterns Used at Mayo Clinic, Jacksonville, Florida, for Patients with Locally Advanced Squamous Cell Carcinoma with Clinical Perineural Invasion. CT indicates computed tomography; MRI, magnetic resonance imaging; and PET-CT, positron emission tomography-computed tomography.

The incorporation of immunotherapy into the management of SCC with CPNI presents a valuable opportunity to enhance multidisciplinary care. While early clinical outcomes are promising, larger randomized trials are needed to confirm the efficacy and safety of immunotherapy in cSCC with cPNI. Further research is needed to identify predictive biomarkers of response, stratify patients by risk and immune status, and optimize therapeutic strategies for diverse populations, including those with severe comorbid conditions or immunosuppression due to organ transplants or other causes. In the meantime, a collaborative, multidisciplinary approach—such as our institution’s framework, which integrates dermatologic, surgical, oncologic, and radiologic expertise—can serve as an effective model for managing complex cases. This framework allows for optimized and tailored sequencing of therapies, balancing immunotherapy, surgery, and RT to maximize patient outcomes and minimize morbidity. Our institutional treatment approach for locally advanced SCC with cPNI is outlined in Figure 4.

## 5. Conclusions

Overall, management of cSCC with cPNI is challenging, requiring nuanced strategies for diagnosis, treatment, and prognosis. While immunotherapy is a promising treatment option, further investigation is needed to define its optimal role, including appropriate sequencing in the treatment algorithm for PNI-associated SCC. Multidisciplinary collaboration is crucial for advancing care and improving outcomes for this high-risk patient population. Our review outlines the current clinical practice patterns at our institution, providing novel insights within the broader landscape of emerging therapeutic approaches for SCC with PNI.

Future directions for immunotherapy and radiation in the treatment of cPNI in cSCC center on integrating immunotherapy into multimodal regimens, optimizing perioperative use, and refining radiation techniques for improved disease control and functional outcomes.

## Figures and Tables

**Table 1:** Literature on Immunotherapy for Clinical Perineural Invasion in Cutaneous Squamous Cell Carcinoma.

Study	No. of Patients	Immunotherapy Used	Response	Follow-Up Time	Overall Survival	Progression-Free Survival	Adverse Effects
Nightingale et al. [21]	13	PD-1 inhibitor	69.2% OR (46.2% CR; 23.1% PR); median time to response of 2.1 months	September 2017 to May 2021	Not reported	23% had a median time to progression of 3.5 months	No grade 3–4 treatment-related adverse events
Khan et al. [19]	121	PD-1 inhibitor	61.2% CLR or stable disease	Not reported	Not reported	Not reported	Not Reported
Cavanagh et al. [18]	20	Cemiplimab in 17 patients, and Pembrolizumab in 3 patients.	70.0% perineural spread response at 5 months; 15.0% pseudoprogression; 5.0% progression	April 2018 to February 2022	18.5 months	Not reported	Not reported
Wu et al. [22]	11	Immune checkpoint inhibitor	81.8% RDC	Not reported	Not reported	Not reported	Not reported
Lopetegui-Lia et al. [20]	12	Cemiplimab or Pembrolizumab	83.3% clinical response; 8.3% CR; 58.3% PR	Median follow-up of 23 months	Not reported	Not reported	Not reported, but 50.0% remained on IO after the study

CLR, complete local response; CR, complete response; IO, immunotherapy; OR, overall response; PR, partial response; RDC, radiologic disease control; PD-1, programmed cell death protein-1.

## Supplementary Materials

The following supporting information can be downloaded at: https://www.mdpi.com/article/10.3390/cancers17243921/s1, Figure S1: Flow diagram of database literature sources using keywords, “clinical perineural invasion AND cutaneous squamous cell carcinoma AND immunotherapy”.

## Abbreviations

The following abbreviations are used in this manuscript:

AJCC	American Joint Committee on Cancer
BWH	Brigham and Women’s Hospital
cPNI	clinical perineural invasion
CT	computed tomography
cSCC	cutaneous squamous cell carcinoma
FDG-PET	fludeoxyglucose F18 positron emission tomography
HNSCC	head and neck squamous cell carcinoma
ICI	immune checkpoint inhibitor
iPNI	incidental perineural invasion
MRI	magnetic resonance imaging
PNI	perineural invasion
PD-1	programmed cell death protein-1
PD-L1	programmed death-ligand 1
RT	radiation therapy
SCC	squamous cell carcinoma

## References

[1] Wysong A. (2023). Squamous-Cell Carcinoma of the Skin. N. Engl. J. Med..

[2] de Jong E., Lammerts M., Genders R., Bavinck J.B. (2021). Update of advanced cutaneous squamous cell carcinoma. J. Eur. Acad. Dermatol. Venereol..

[3] Hirotsu K.E., Aasi S.Z., Samson K.K., Zheng C., Nazaroff J.R., Voller L.M., Ruiz E.S., Ran N.A., Granger E.E., Koyfman S.A. (2025). Lymphovascular invasion is an independent predictor of metastasis and disease-specific death in cutaneous squamous cell carcinoma: A multicenter retrospective study. J. Am. Acad. Dermatol..

[4] Zeng S., Fu L., Zhou P., Ling H. (2020). Identifying risk factors for the prognosis of head and neck cutaneous squamous cell carcinoma: A systematic review and meta-analysis. PLoS ONE.

[5] Massey P.R., Wang D.M., Murad F., Mulvaney P., Moore K., Okhovat J.-P., Russell-Goldman E., Lin W.M., Piris A., Huilgol S.C. (2023). Extensive Perineural Invasion vs. Nerve Caliber to Assess Cutaneous Squamous Cell Carcinoma Prognosis. JAMA Dermatol..

[6] Alam M., Armstrong A., Baum C., Bordeaux J.S., Brown M., Busam K.J., Eisen D.B., Iyengar V., Lober C., Margolis D.J. (2018). Guidelines of care for the management of cutaneous squamous cell carcinoma. J. Am. Acad. Dermatol..

[7] Chow L.Q.M. (2020). Head and Neck Cancer. N. Engl. J. Med..

[8] Balamucki C.J., Mancuso A.A., Amdur R.J., Kirwan J.M., Morris C.G., Flowers F.P., Stoer C.B., Cognetta A.B., Mendenhall W.M. (2012). Skin carcinoma of the head and neck with perineural invasion. Am. J. Otolaryngol..

[9] Holtzman A.L., Mendenhall W.M. (2020). High-dose conformal proton therapy for clinical perineural invasion in cutaneous head and neck cancer. Oral Oncol..

[10] Karia P.S., Morgan F.C., Ruiz E.S., Schmults C.D. (2017). Clinical and Incidental Perineural Invasion of Cutaneous Squamous Cell Carcinoma. JAMA Dermatol..

[11] Karia P.S., Jambusaria-Pahlajani A., Harrington D.P., Murphy G.F., Qureshi A.A., Schmults C.D. (2014). Evaluation of American Joint Committee on Cancer, International Union Against Cancer, and Brigham and Women’s Hospital Tumor Staging for Cutaneous Squamous Cell Carcinoma. J. Clin. Oncol..

[12] Frydenlund N., Leone D.A., Mitchell B., Abbas O., Dhingra J., Mahalingam M. (2015). Perineural invasion in cutaneous squamous cell carcinoma: Role of immunohistochemistry, anatomical site, and the high-affinity nerve growth factor receptor TrkA. Hum. Pathol..

[13] Morandi E.M., Rauchenwald T., Puelzl P., Zelger B.W., Zelger B.G., Henninger B., Pierer G., Wolfram D. (2021). Hide-and-seek: Neurotropic squamous cell carcinoma of the periorbital region—A series of five cases and review of the literature. J. Dtsch. Dermatol. Ges..

[14] Misztal C.I., Green C., Mei C., Bhatia R., Torres J.M.V., Kamrava B., Moon S., Nicolli E., Weed D., Sargi Z. (2021). Molecular and Cellular Mechanisms of Perineural Invasion in Oral Squamous Cell Carcinoma: Potential Targets for Therapeutic Intervention. Cancers.

[15] Haug K., Breuninger H., Metzler G., Eigentler T., Eichner M., Häfner H.-M., Schnabl S.M. (2020). Prognostic Impact of Perineural Invasion in Cutaneous Squamous Cell Carcinoma: Results of a Prospective Study of 1,399 Tumors. J. Investig. Dermatol..

[16] Gupta A., Veness M., De’AMbrosis B., Selva D., Huilgol S.C. (2015). Management of squamous cell and basal cell carcinomas of the head and neck with perineural invasion. Australas. J. Dermatol..

[17] Shalhout S.Z., Emerick K.S., Kaufman H.L., Miller D.M. (2021). Immunotherapy for Non-melanoma Skin Cancer. Curr. Oncol. Rep..

[18] Cavanagh K., McLean L.S., Lim A.M., Cardin A., Levy S.M., Rischin D. (2024). Assessment of perineural spread in advanced cutaneous squamous cell carcinomas treated with immunotherapy. Cancer Imaging.

[19] Khan M., Xenopoulou D., Khachemoune A. (2022). Systematic review on outcomes of the use of adjuvant pharmacotherapy for treatment of cutaneous malignancies exhibiting perineural invasion: Promising efficacy of anti-PD1 therapy. Arch. Dermatol. Res..

[20] Lopetegui-Lia N., Dima D., Buchberger D.S., Yalamanchali A., Osantowski B., Ondeck M., Lorenz R.R., Prendes B., Ku J., Lamarre E. (2023). Immunotherapy response in patients with cutaneous squamous cell carcinoma of head and neck with cranial nerve involvement. Head Neck.

[21] Nightingale J., Gandhi M., Helena J., Bowman J., McGrath M., Coward J., Porceddu S., Ladwa R., Panizza B. (2022). Immunotherapy for the treatment of perineural spread in cutaneous head and neck squamous cell carcinoma: Time to rethink treatment paradigms. Head Neck.

[22] Wu M.P., Reinshagen K.L., Cunnane M.B., Shalhout S.Z., Kaufman H.L., Miller D., Emerick K.S. (2021). Clinical Perineural Invasion and Immunotherapy for Head and Neck Cutaneous Squamous Cell Carcinoma. Laryngoscope..

[23] Rischin D., Porceddu S., Day F., Brungs D.P., Christie H., Jackson J.E., Stein B.N., Su Y.B., Ladwa R., Adams G. (2025). Adjuvant Cemiplimab or Placebo in High-Risk Cutaneous Squamous-Cell Carcinoma. N. Engl. J. Med..

[24] Gross N.D., Miller D.M., Khushalani N.I., Divi V., Ruiz E.S., Lipson E.J., Meier F., Su Y.B., Swiecicki P.L., Atlas J. (2022). Neoadjuvant Cemiplimab for Stage II to IV Cutaneous Squamous-Cell Carcinoma. N. Engl. J. Med..

[25] Wilde D.C., Glaun M.E., Wong M.K., Gross N.D. (2023). Neoadjuvant Approaches to Non-Melanoma Skin Cancer. Cancers.

[26] Linedale R., Schmidt C., King B.T., Ganko A.G., Simpson F., Panizza B.J., Leggatt G.R. (2017). Elevated frequencies of CD8 T cells expressing PD-1, CTLA-4 and Tim-3 within tumour from perineural squamous cell carcinoma patients. PLoS ONE.

[27] Stevenson M.L., Wang C.Q.F., Abikhair M., Roudiani N., Felsen D., Krueger J.G., Pavlick A.C., Carucci J.A. (2017). Expression of Programmed Cell Death Ligand in Cutaneous Squamous Cell Carcinoma and Treatment of Locally Advanced Disease with Pembrolizumab. JAMA Dermatol..

[28] Kuo Y.-J., Gide T.N., Mao Y., Adegoke N.A., Bennett T., Menzies A.M., Long G.V., Wilmott J.S., da Silva I.E.D.P. (2025). Multiomic analysis of cutaneous squamous cell carcinoma (cSCC) and association with response to anti-PD1 therapy (PD1). J. Clin. Oncol..

[29] Arnon J., Dinstag G., Chayen B., Tirosh O., Kinar Y., Ben-Zvi D.S., Beker T., Elia A., Pikarsky E., Yakir R. (2025). Predication of clinical outcomes of advanced cutaneous squamous cell carcinoma to PD1 inhibition directly from histopathology slides using inferred transcriptomics. J. Clin. Oncol..

[30] Bryant C.M., Dagan R., Holtzman A.L., Fernandes R., Bunnell A., Mendenhall W.M. (2021). Passively Scattered Proton Therapy for Nonmelanoma Skin Cancer with Clinical Perineural Invasion. Int. J. Part. Ther..

[31] Ferini G., Palmisciano P., Forte S., Viola A., Martorana E., Parisi S., Valenti V., Fichera C., Umana G.E., Pergolizzi S. (2022). Advanced or Metastatic Cutaneous Squamous Cell Carcinoma: The Current and Future Role of Radiation Therapy in the Era of Immunotherapy. Cancers.

[32] Manukian G., Bar-Ad V., Lu B., Argiris A., Johnson J.M. (2019). Combining Radiation and Immune Checkpoint Blockade in the Treatment of Head and Neck Squamous Cell Carcinoma. Front. Oncol..

[33] Dovedi S.J., Adlard A.L., Lipowska-Bhalla G., McKenna C., Jones S., Cheadle E.J., Stratford I.J., Poon E., Morrow M., Stewart R. (2014). Acquired Resistance to Fractionated Radiotherapy Can Be Overcome by Concurrent PD-L1 Blockade. Cancer Res..

[34] Kut C., Quon H., Chen X.S. (2024). Emerging Radiotherapy Technologies for Head and Neck Squamous Cell Carcinoma: Challenges and Opportunities in the Era of Immunotherapy. Cancers.

[35] Karam S.D., Raben D. (2019). Radioimmunotherapy for the treatment of head and neck cancer. Lancet Oncol..

[36] Baumann B.C., Mitra N., Harton J.G., Xiao Y., Wojcieszynski A.P., Gabriel P.E., Zhong H., Geng H., Doucette A., Wei J. (2020). Comparative Effectiveness of Proton vs. Photon Therapy as Part of Concurrent Chemoradiotherapy for Locally Advanced Cancer. JAMA Oncol..

[37] Bortfeld T.R., Loeffler J.S. (2017). Three ways to make proton therapy affordable. Nature.

[38] Durante M., Formenti S. (2019). Harnessing radiation to improve immunotherapy: Better with particles?. Br. J. Radiol..

[39] Hartmann L., Schröter P., Osen W., Baumann D., Offringa R., Moustafa M., Will R., Debus J., Brons S., Rieken S. (2020). Photon versus carbon ion irradiation: Immunomodulatory effects exerted on murine tumor cell lines. Sci. Rep..

[40] Liu S., He X., Liang S., Wu A., Liu L., Hu W. (2025). Carbon ion irradiation mobilizes antitumor immunity: From concept to the clinic. Radiat. Oncol..

[41] Libson K.B., Sheridan C.B., Carr D.R., Shahwan K.T. (2024). Use of Imaging in Cutaneous Squamous Cell Carcinoma to Detect High-Risk Tumor Features, Nodal Metastasis, and Distant Metastasis: A Systematic Review. Dermatol. Surg..

[42] Arya S., Rane P., Deshmukh A. (2014). Oral cavity squamous cell carcinoma: Role of pretreatment imaging and its influence on management. Clin. Radiol..

[43] Overfield C.J., Rhyner P.A., Hall M.R., Bhatt A.A. (2024). More than Skin Deep: Imaging of Dermatologic Disease in the Head and Neck. RadioGraphics.

[44] Majoie C.B., Hulsmans F.-J.H., Verbeeten B., Castelyns J.A., Oldenburger F., Schouwenburg P.F., Bosch D. (1997). Perineural tumor extension along the trigeminal nerve: Magnetic resonance imaging findings. Eur. J. Radiol..

[45] Paes F.M., Singer A.D., Checkver A.N., Palmquist R.A., De La Vega G., Sidani C. (2013). Perineural Spread in Head and Neck Malignancies: Clinical Significance and Evaluation with ^18^F-FDG PET/CT. RadioGraphics.

[46] Wei A.H., Cassard L., Fan C., Seck S., Vazquez M.R., Bena J., Stultz T., Koyfman S.A., Vidimos A.T. (2025). Radiologic imaging aids management of high-risk cutaneous squamous cell carcinoma: A retrospective cohort study. J. Am. Acad. Dermatol..

[47] Agarwal M., Wangaryattawanich P., Rath T.J. (2019). Perineural Tumor Spread in Head and Neck Malignancies. Semin. Roentgenol..

[48] Aiken A.H., Rath T.J., Anzai Y., Branstetter B.F., Hoang J.K., Wiggins R.H., Juliano A.F., Glastonbury C., Phillips C.D., Brown R. (2018). ACR Neck Imaging Reporting and Data Systems (NI-RADS): A White Paper of the ACR NI-RADS Committee. J. Am. Coll. Radiol..

[49] Fulcher C.D., Haigentz M., Ow T.J. (2017). The Education Committee of the American Head and Neck Society (AHNS) AHNS Series: Do you know your guidelines? Principles of treatment for locally advanced or unresectable head and neck squamous cell carcinoma. Head Neck.

[50] Digonnet A., Hamoir M., Andry G., Haigentz M., Takes R.P., Silver C.E., Hartl D.M., Strojan P., Rinaldo A., de Bree R. (2012). Post-therapeutic surveillance strategies in head and neck squamous cell carcinoma. Eur. Arch. Oto-Rhino-Laryngol..

[51] Granger E.E., Ran N.A., Groover M.K., Koyfman S.A., Vidimos A.T., Wysong A., Carr D.R., Shahwan K.T., Hirotsu K.E., Carucci J.A. (2024). Most cutaneous squamous cell carcinoma recurrences occur in the first 3 years after diagnosis: A multicenter retrospective cohort study. J. Am. Acad. Dermatol..

