# Supplementary material for: Immunotherapy and Radiation for Clinical Perineural Invasion in Cutaneous Squamous Cell Carcinoma
Source: Cancers (Basel). 2025 Dec 8;17(24):3921. doi: 10.3390/cancers17243921 (PMC12730315; doi:10.3390/cancers17243921)
Supplement: Supplementary file 1 [file cancers-17-03921-s001.zip › cancers-3968616-supplementary.pdf]

cancers

MDPI

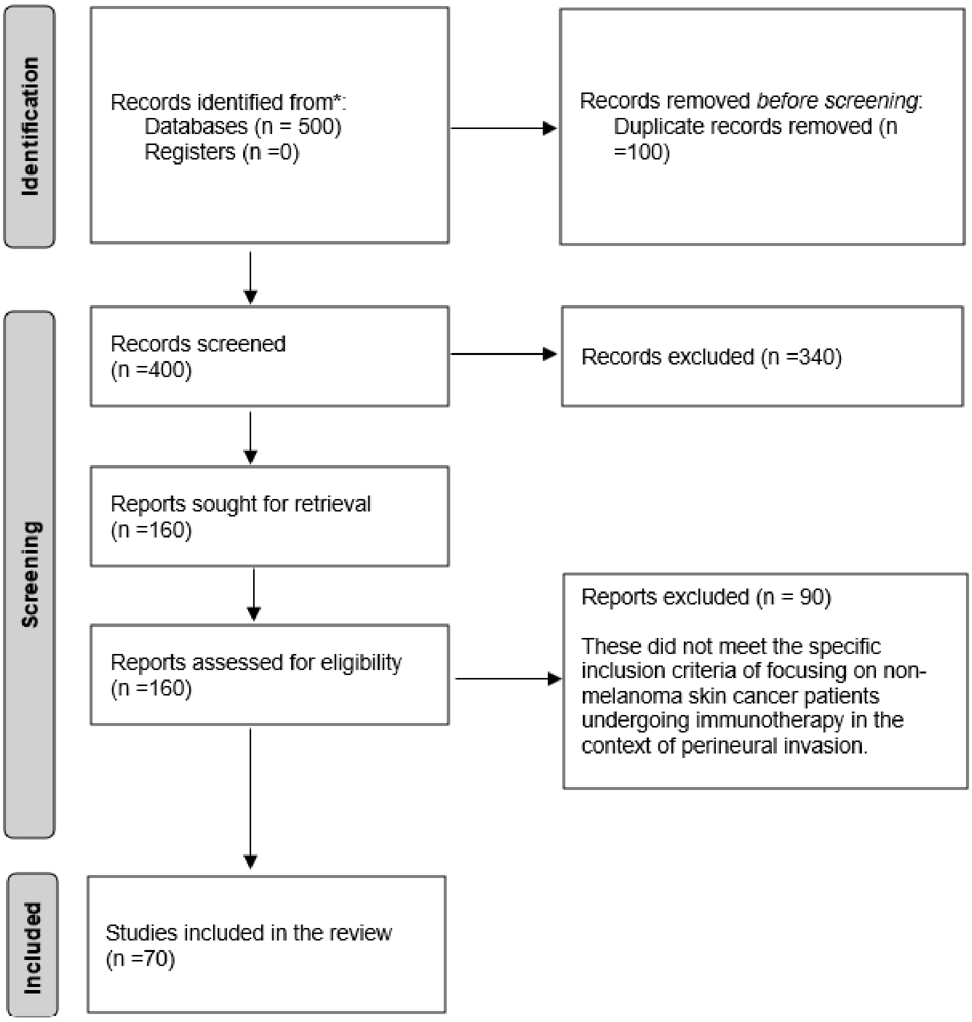

Supplemental Figure S1. Flow diagram of database literature sources using keywords, "clinical perineural invasion AND cutaneous squamous cell carcinoma AND immunotherapy". ***.** Databases searched included PubMed, Google Scholar, Ovid, and Scopus.
